# High internal radiation exposure associated with low socio-economic status six years after the Fukushima nuclear disaster: A case report

**DOI:** 10.1097/MD.0000000000017989

**Published:** 2019-11-22

**Authors:** Toyoaki Sawano, Toshiyuki Kambe, Yuki Seno, Ran Konoe, Yoshitaka Nishikawa, Akihiko Ozaki, Yuki Shimada, Tomohiro Morita, Hiroaki Saito, Masaharu Tsubokura

**Affiliations:** a Department of Surgery, Minamisoma Municipal General Hospital; b Department of Public Health, Fukushima Medical University School of Medicine; c Department of Pulmonary Medicine, Minamisoma Municipal General Hospital, Fukushima, Japan; d Faculty of Medicine, Comenius University, Bratislava, Slovakia; e Department of Internal Medicine, Soma Central Hospital, Fukushima; f Department of Health Informatics, School of Public Health, Kyoto University, Kyoto; g Research Center for Community Health, Minamisoma Municipal General Hospital, Minamisoma; h Department of Breast Surgery, Jyoban Hospital of Tokiwa Foundation; i Department of Neurosurgery, Minamisoma Municipal General Hospital, Fukushima; j Department of Gastroenterology, Sendai Kousei Hospital, Miyagi, Japan.

**Keywords:** case report, internal radiation contamination, nuclear disasters, social support, vulnerable groups

## Abstract

**Rationale:**

Managing the health of vulnerable groups is an important component of health care. Given the long-term burden of radiation-release incidents among those exposed, managing the health of vulnerable groups following a nuclear disaster is very important. However, there is limited information available concerning the long-term management of the health effects of radiation exposure in vulnerable groups following nuclear disasters. After the Fukushima Daiichi Nuclear Power Plant (FDNPP) accident, Minamisoma City launched internal radiation exposure monitoring program for local residents, using whole body counter (WBC) units. In 2017, a man of low socio-economic status (SES), was found to have the highest level of internal contamination detected in a person living in the Soma District in recent years. This report describes the case so that the lessons learned can be applied in future nuclear disaster settings.

**Patient concerns:**

A 77-year-old Japanese man, who had been homeless for 2 months and had been staying in the exclusion zone of Minamisoma City, was brought to our hospital. He had become homeless because a lack of communication between social support services had led to his eviction from leased housing after free housing support for evacuees was terminated.

**Diagnoses:**

He was admitted with a diagnosis of dehydration and malnutrition. A WBC unit was used to assess his body burden of radioactive cesium. This revealed levels of Cs-134 and Cs-137 of 538 Bq/body and 4,993 Bq/body, respectively.

**Intervention:**

He received intravenous fluid therapy and health monitoring. The paperwork required for him to receive public income support was processed during hospitalization.

**Outcome:**

He was discharged to public housing after 9 days, and municipal workers started visiting him regularly after his discharge.

**Lessons:**

A high level of internal radiation contamination may occur after a nuclear disaster. This may be associated with a decline in social support, poverty, and social isolation, and may have more impact on people in poor health than on the general population. It would be useful to strengthen linkages between local government and welfare service providers to increase social support for vulnerable groups requiring health care, not only following disasters, but also under normal circumstances.

## 1 Introduction

Managing the health of vulnerable groups is important in the context of significant disparities in access to healthcare.^[1–4]^ Studies have shown that social determinants of health have a large impact on health outcomes, and people with low socio-economic status (SES) are particularly vulnerable following hazardous events such as natural disasters.^[1,5]^ Members of vulnerable groups often have difficulty in extricating themselves from unfavorable conditions for socio-economic reasons.^[4–6]^ One of the most important issues to address, is improving access to social support services for vulnerable people as part of post-disaster health management.

Given the long-term burden of radiation-release incidents among people living in disaster-stricken areas, managing the health of vulnerable groups by providing access to healthcare is very important following nuclear disasters. Previous studies have shown that evacuation after disasters has a greater impact on vulnerable groups such as the elderly and the disabled, than on the general population.^[7]^ The implementation of emergency evacuation to prevent the direct health effects of radiation in the period directly after the disaster, and long-term management of the chronic disease during the post-disaster period are both imperative for health management of vulnerable groups.^[8]^ However, there is limited information available concerning the long-term management of the health effects of radiation exposure in vulnerable groups following nuclear disasters.

In March 2011, the government of Japan issued a mandatory evacuation order following the Fukushima Daiichi Nuclear Power Plant (FDNPP) accident which followed a major earthquake and tsunami on March 11, 2011. The government of Fukushima Prefecture offered rooms to the people evacuated after the FDNPP accident. However, in March 2017, the government decided to terminate the free housing support for evacuees who had lived outside the mandatory evacuation zone in Fukushima Prefecture before the disaster. In December 2018, approximately 43,000 evacuated residents were still displaced and living in other areas in and around the Fukushima Prefecture. Vulnerable people were among the evacuated residents.^[9]^ Although the health effects of radiation exposure caused by FDNPP accident are reported to have been negligible,^[10–12]^ implementing countermeasures to minimize long-term radiation exposure is still imperative for public health. Particular attention needs to be paid to managing internal radiation exposure. Because it occurs over a prolonged period, and depends on individual lifestyles, it is difficult to identify people at high risk of internal radiation exposure.^[13]^ While high levels of internal contamination were observed among a portion of the population after the nuclear accident,^[14,15]^ limited information is available regarding the risk factors for internal radiation contamination at an individual level.^[13]^

In response to the FDNPP accident, Minamisoma City launched a voluntary internal radiation exposure monitoring program for local residents in July 2011, using Whole Body Counter (WBC) units at Minamisoma Municipal General Hospital, a core medical institution in the area affected by FDNPP accident. All children in Fukushima Prefecture were screened, while internal radiation screening of adults was voluntary.^[16]^ In 2017, 6 years after the nuclear accident, a man of low SES was found to have the highest level of internal contamination detected in a person living in Soma District (Soma and Minamisoma cities) in recent years. Our case findings may contribute to the development of necessary countermeasures to prevent internal radiation contamination after nuclear accidents. We therefore, consider it important to publish a detailed case report, and to reveal the sequence of events which led to this man's health problems.

## 2 Case presentation

In August 2017, a 77-year-old Japanese man who had been homeless for 2 months and had been staying in the exclusion zone of Minamisoma City, was brought to the emergency department of our hospital by a police officer. In the emergency room, he was found to have dehydration and malnutrition. He was admitted so that he could receive intravenous rehydration and health monitoring.

**Figure 1:**
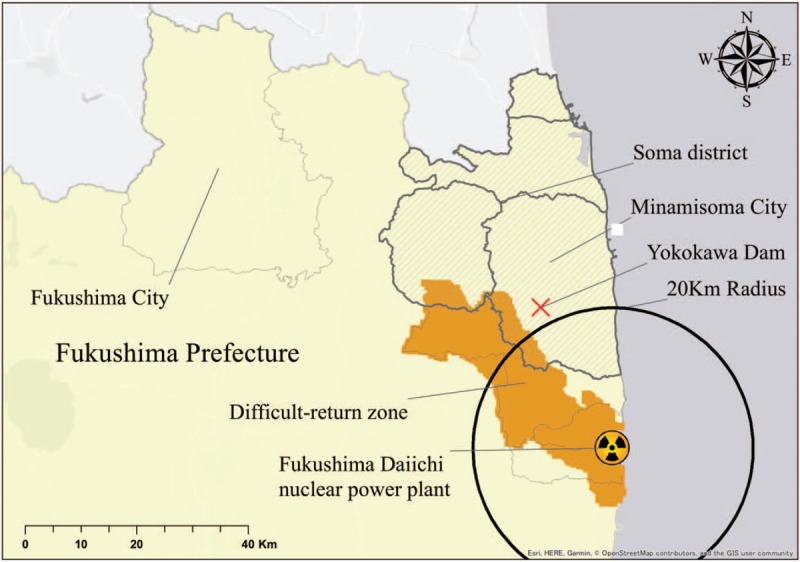
Map showing the location of the places named in this report in relation to the Fukushima Daiichi Nuclear Power Plant which was the source of the nuclear disaster and the exclusion zone, namely difficult-to-return area zone by the Japanese government.

Prior to the disaster, he had lived in the coastal area of Minamisoma City until his house was swept away by the tsunami on March 11, 2011. Although he was reported to have been diagnosed with a psychological disorder before the disaster, no further details were available. He reported that he had lived in free leased housing in Fukushima City provided to evacuees after the FDNPP accident. As his house before the disaster was not within the mandatory evacuation zone, he was forced to leave this housing in July 2017 when the free housing support provided by the local government was terminated. Although social support, including public income support, is available to the poor in Japan, he had not been provided with new accommodation because of a lack of communication between social support services. He did not have sufficient funds to rent a new home and this had led him to become homeless. After his eviction, he lived in a cave in the mountain district of Minamisoma City, near the Yokokawa Dam (Fig. 1). The mountain area surrounding Yokokawa Dam was an area with relatively high level of ambient dose equivalent, and was designated as an exclusion zone because the annual dose from external radiation was estimated to be at least 50 mSv/year in 2017. As he did not have enough money to buy food, he survived by drinking stream water and eating mushrooms, wild plants, and fresh-water fish found in the area.

On examination, he was diagnosed with sarcopenia due to malnutrition, and psychological disease. His physical examination on admission showed no signs of radiation exposure. His total body burden of radioactive cesium (Cesium; Cs-134 and Cs-137) was measured using a stand-type WBC unit (Fastscan Model 2251; Mirion Technologies (Canberra), Inc, Meriden, CT) and he was found to have levels of Cs-134 and Cs-137 of 538 Bq/body and 4993 Bq/body respectively. The annual effective dose from internal radiation, calculated using his Cs-134 and Cs-137 results, was 0.20 mSv/year, assuming that the amount of Cs activity detected by the WBC examination was in an equilibrium state between ingestion and excretion throughout 1 year.

The paperwork required for public income support was processed during his hospitalization. Nine days after admission, after his condition had improved, he was discharged to a new home provided as public housing. With the aid of social support, municipal workers started visiting him regularly after his discharge.

## 3 Discussion

This is a case of a 77-year-old man who had become homeless due to a lack of social support, after the free housing support for people evacuated after the 2011 nuclear disaster was terminated in 2017. He was found to have a high level of internal contamination on admission. This case highlights that high levels of internal contamination after nuclear disaster may be related to low SES.

This man's total body burden of radioactive cesium was the highest level of internal radiation contamination found in people who had WBC screening in Minamisoma Municipal General Hospital in 2017, and was also the highest level of internal radiation contamination that has been found using WBC in Soma District. Based on previously-published research on internal radiation contamination,^[10]^ we hypothesize that ingestion of mushrooms, wild-plants, and fresh-water fish found in the exclusion zone, rather than inhalation or initial radiation exposure, explains his high total body burden of radioactive cesium.

The maximum internal contamination detected following the FDNPP accident has gradually decreased year-by-year. The maximum levels of Cs-134 and Cs-137 reported were 6713 Bq/body and 10730 Bq/body, respectively, in Minamisoma City in 2012,^[15]^ compared to the levels of Cs-134 and Cs-137 detected were 538 Bq/body and 4993 Bq/body, respectively, in this case. Nevertheless, the internal contamination level detected in this case was the third highest level detected in Minamisoma City since 2013.

High internal radiation contamination after nuclear disasters may be associated with a decline in social support, leading to poverty and social isolation. A lack of social support has a greater effect on people in a poor state of health than on those in the general population. Minimizing the level of internal and external radiation exposure among residents is a crucial component of health management after a nuclear disaster. Thus, it is important to determine factors that increase the level of radiation contamination after a nuclear disaster.

While external exposure of individuals depends on the level of ambient dose equivalent at the locations where they spend the most time, internal radiation contamination depends on individual lifestyle, and little is known about the types of people who are at high risk of developing high levels of internal radiation contamination. Previous studies have found that several factors, such as dietary preferences, avoidance of local products and restriction of shipment of dietary items, contributed to the level of internal radiation contamination among residents.^[10]^ In this case, however, the man had been forced to eat contaminated foods because of a lack of financial and social support, and not because of his dietary preferences.

In addition to dietary factors, a decrease in the level of social support provided to people of low SES, may contribute to high levels of internal radiation contamination after a nuclear disaster, as was seen in this case.^[17]^ In this context, it may be worthwhile for local governments to increase social support for vulnerable groups to enable them to access healthcare by strengthening linkages between local government and local welfare service providers, not only following disasters but also under normal circumstances.

Social factors are more important contributors to high levels of internal radiation contamination in the chronic phase of a nuclear disaster than in the acute phase, although both the level of ambient dose equivalent, and the radiological contamination level of foods decrease over time. It may be worthwhile to focus on ensuring that vulnerable groups have access to health care, in order to minimize the health effects of internal radiation contamination after a nuclear disaster.

The health impacts of nuclear disasters on residents are not restricted to the effects of radiation exposure, but are also caused by the indirect impacts. In vulnerable groups, factors, such as evacuation and deterioration of social relationships, including collapse of the community, may cause nuclear disasters to have be more significant health impact than that of other natural disasters. In this case, patient-specific factors, such as a psychological disorder, social isolation, and old age, contributed significantly to the health consequences.

It has also been shown that social issues such as poor access to medical treatment, the increased numbers of people requiring social welfare and nursing care, and a shortage of human resources for health can have a large health impact in disaster-stricken areas following other natural disasters.^[18]^ However, the effects of radiation exposure can persist for a longer period than the effects of other types of natural disasters.^[19]^ It is therefore necessary to acknowledge that, in addition to support during the acute phase of the disaster, long-term social support for vulnerable groups is crucial for post-disaster management of health issues.

## 4 Conclusion

This report highlights the need to provide long-term social support to vulnerable groups following nuclear disasters, in addition to taking measures to minimize the health effects of high levels of radiation exposure.

## Acknowledgments

We would like to thank Editage (www.editage.jp) for English language editing, and also thank Masatsugu Tanaki for his constructive opinions on this study.

## Author contributions

**Conceptualization:** Toyoaki Sawano.

**Resources:** Toyoaki Sawano, Toshiyuki Kambe, Ran Konoe.

**Supervision:** Toshiyuki Kambe, Yoshitaka Nishikawa, Akihiko Ozaki, Yuki Shimada, Tomohiro Morita, Hiroaki Saito, Masaharu Tsubokura.

**Validation:** Toyoaki Sawano.

**Writing – original draft:** Toyoaki Sawano.

**Writing – review & editing:** Yuki Seno, Masaharu Tsubokura.

Toyoaki Sawano orcid: 0000-0002-1482-6618.
